# Multi-risk factors joint prediction model for risk prediction of retinopathy of prematurity

**DOI:** 10.1007/s13167-024-00363-7

**Published:** 2024-05-09

**Authors:** Shaobin Chen, Xinyu Zhao, Zhenquan Wu, Kangyang Cao, Yulin Zhang, Tao Tan, Chan-Tong Lam, Yanwu Xu, Guoming Zhang, Yue Sun

**Affiliations:** 1 Faculty of Applied Sciences, Macao Polytechnic University, Gomes Street, Macao, China; 2 Department of Electrical Engineering, Eindhoven University of Technology, Eindhoven, 5612 AP The Netherlands; 3 Shenzhen Eye Hospital, Jinan University, Shenzhen Eye Institute, Shenzhen, 518040 China; 4 School of Future Technology, South China University of Technology, Guangzhou, Guangzhou; Pazhou Lab, China

**Keywords:** Retinopathy of prematurity, Infant, Biometric information, Early screening, Risk of blindness, Predictive, Preventive, Personalized medicine (PPPM / 3 PM), Artificial intelligence

## Abstract

**Purpose:**

Retinopathy of prematurity (ROP) is a retinal vascular proliferative disease common in low birth weight and premature infants and is one of the main causes of blindness in children.

In the context of predictive, preventive and personalized medicine (PPPM/3PM), early screening, identification and treatment of ROP will directly contribute to improve patients’ long-term visual prognosis and reduce the risk of blindness. Thus, our objective is to establish an artificial intelligence (AI) algorithm combined with clinical demographics to create a risk model for ROP including treatment-requiring retinopathy of prematurity (TR-ROP) infants.

**Methods:**

A total of 22,569 infants who underwent routine ROP screening in Shenzhen Eye Hospital from March 2003 to September 2023 were collected, including 3335 infants with ROP and 1234 infants with TR-ROP among ROP infants. Two machine learning methods of logistic regression and decision tree and a deep learning method of multi-layer perceptron were trained by using the relevant combination of risk factors such as birth weight (BW), gestational age (GA), gender, whether multiple births (MB) and mode of delivery (MD) to achieve the risk prediction of ROP and TR-ROP. We used five evaluation metrics to evaluate the performance of the risk prediction model. The area under the receiver operating characteristic curve (AUC) and the area under the precision-recall curve (AUCPR) were the main measurement metrics.

**Results:**

In the risk prediction for ROP, the BW + GA demonstrated the optimal performance (mean ± SD, AUCPR: 0.4849 ± 0.0175, AUC: 0.8124 ± 0.0033). In the risk prediction of TR-ROP, reasonable performance can be achieved by using GA + BW + Gender + MD + MB (AUCPR: 0.2713 ± 0.0214, AUC: 0.8328 ± 0.0088).

**Conclusions:**

Combining risk factors with AI in screening programs for ROP could achieve risk prediction of ROP and TR-ROP, detect TR-ROP earlier and reduce the number of ROP examinations and unnecessary physiological stress in low-risk infants. Therefore, combining ROP-related biometric information with AI is a cost-effective strategy for predictive diagnostic, targeted prevention, and personalization of medical services in early screening and treatment of ROP.

## Introduction

Retinopathy of Prematurity (ROP) is a pathological process that occurs in the immature retinal tissue of low birth weight preterm infants [1–3]. It is a developmental disorder of retinal vessel that can develop into tractional retinal detachment in the later stage, which may lead to vision loss and is one of the leading cause of blindness in infants [4]. According to a retrospective analysis [5], ROP caused as much as 8% of blindness in children worldwide. During the first epidemic of ROP in the mid-twentieth century, the main risk factor at the time were unmonitored supplemental oxygen, the use of high concentration of oxygen and unrestricted oxygen therapy to improve survival of preterm infants, mainly affecting preterm infants in industrialized countries such as the United States and Western Europe [3, 6]. In the following two decades, as oxygen use in newborns was controlled, the blindness of ROP declined and the survival rate of extremely preterm infants greatly improved, and a second epidemic of ROP emerged in the industrialized countries in the 1970s [7]. With the widespread promotion of monitored oxygen inhalation for preterm infants, ROP screening and treatment have been popularized. In particular, developed countries have formulated various systems to strictly control the blinding rate of ROP, which has stabilized the incidence of ROP. Nevertheless, due to large differences between countries in preterm birth rates, the level of neonatal intensive care, the number of trained neonatal nurses, and the effectiveness of screening and treatment programs for ROP [8], blindness due to ROP varied widely among countries, especially with higher occurrence of severe ROP in preterm infants in low- and middle-income countries [9]. Since then, the epidemiology of ROP in preterm infants has changed both geographically and temporally, primarily related to two major factors: a progressively lower neonatal mortality rate and the implementation of stringent oxygen monitoring protocols [10, 11].

To mitigate the risk of vision loss caused by ROP, there are the following two prevention strategies: (1) Primary prevention of ROP, through strict oxygen titration, effectively reduces the incidence of treatment-requiring retinopathy of prematurity (TR-ROP) [12]; (2) Secondary prevention of ROP, providing a higher proportion of oxygen, and implementing timely ROP screening in the neonatal intensive care units (NICUs) for all at-risk newborns [13]. However, there is a delicate balance: a lower ratio of oxygen is effective in reducing all subsequent morbidities in preterm infants but thus increases the probability of mortality, and vice versa [14]. ROP screening was recommended for preterm infants with gestational age (GA) < 31 weeks or birth weight (BW) < 1501 g based on demographic criteria in the United States [12], whereas the range of BW and GA of affected infants was much broader in low- and middle-income countries [8, 15, 16]. ROP Examinations should be scheduled based on the preterm infant’s gestational age at birth and the presence and severity of subsequent disease [1]. ROP screening helps identify preterm infants who progress to TR-ROP so that timely treatment can be provided. Over the past few decades, the incidence of blindness due to ROP had shown a rapid increase in many low- and middle-income countries. Many NICUs in these countries lacked the material resources required for primary prevention of ROP, such as oxygen blenders and pulse oxygenation monitors [9], which further contributed to higher incidence and severity of ROP in these countries [17], with even mildly preterm infants at risk for blinding ROP, and therefore, more infants needing to be screened [1, 18]. As emphasized in the European association for predictive, preventive and personalized Medicine (EPMA) white paper [19], identifying infants at high risk of ROP is an crucial step in preventing blindness due to ROP.

### Working hypothesis and study aims in the PPPM framework

Artificial intelligence (AI) algorithms were already widely used to assist in the diagnosis of ophthalmic diseases, which will be able to ease the burden to some extent in countries or regions where the burden of these diseases far exceeds existing screening capabilities [20, 21]. The diagnosis of ROP involved three main clinical features: Zone, indicating the location of retinal vascularization that provides an indication of infant maturity and the risk of ROP development; Stage, describing the characteristics when acute ROP vascular features appear at the vascular-avascular juncture; and plus disease [4], where retinal vessels exhibit dilation and tortuosity [4, 12, 22]. Deep learning algorithms based on imaging and informatics for ROP primarily focus on the three main clinical characteristics mentioned above, including automatic diagnosis of plus disease [23], quantitative assessment of retinal vascular abnormalities [24, 25], and automatic diagnosis and staging of ROP [26–28]. However, these studies had predominantly utilized imaging data to achieve automatic diagnosis of ROP-related diseases, but few studies had combined the ROP risk factors to build ROP prediction models, which are crucial for the prediction and screening of ROP [29]. Even though some studies had combined two major risk factors such as BW and GA for risk prediction of ROP [13], they had ignored the exploration of crucial risk factors such as mode of delivery (MD), gender and multiple births (MB) [29]. Beyond this, an AI algorithm that showed high diagnostic accuracy in a particular research dataset or in a particular demographic population may not be generalizable to other populations [30]. To close this knowledge gap, we utilized five ROP-related risk factors, including BW, GA, MD, gender, and MB, to explore whether effective ROP risk and TR-ROP risk prediction can be constructed solely based on biometric information. In this study, we retrospectively evaluated the risk prediction performance of AI-based ROP and TR-ROP risk prediction models in a real-world dataset from the Shenzhen Eye Hospital informatics ROP cohort study. We further evaluated the hypothesis that risk prediction models based solely on biometric information can be used to predict the risk of ROP and TR-ROP, aiming to assess potential differences in neonatal care within this population. Therefore, this study aimed to explore the relationship between the biometric information of preterm infants and the risk of ROP and TR-ROP, and to explore the feasibility of the PPPM concept in mitigating blindness caused by ROP in Chinese preterm infants.

## Methods

### Dataset preparation and analysis

This study had received approval from the Medical Ethics Committee of Shenzhen Eye Hospital (SZEH) and was conducted in accordance with the principles of the Helsinki Declaration. Written informed consent had been obtained from the parents of infants when they come to the clinic for ROP screening. As part of the informatics Retinopathy of Prematurity (i-ROP) cohort study, ROP screening was conducted on 22,569 unique patients (BW < 1533 g or GA < 32 weeks) between March 2003 and September 2023. The final diagnostic results were consistently determined by three ROP experts using the comprehensive international classification of ROP (ICROP) standards [4, 12]. Statistical single-factor analysis was performed using IBM SPSS Statistics 25 software for both ROP and TR-ROP risk prediction, considering five candidate risk factors: BW, GA, gender, MB, and MD (eutocia or caesarean section). For continuous variables such as BW and GA, Mann-Whitney U test [31] was employed in the single-factor analysis, while for categorical variables such as gender, MB, and MD, Chi-Squared test was applied. Two-side *p* values < 0.05 were considered statistically significant. As can be seen from Table 1, in the risk prediction for both ROP and TR-ROP, four factors, namely BW (*p* < 0.001, *p* < 0.001), GA (*p* < 0.001, *p* < 0.001), gender (*p* < 0.001, *p* < 0.001), and MD (*p* < 0.001, *p* < 0.001) exhibited significant statistical differences (*p* < 0.05), while MB (*p* = 0.243, *p* = 0.878) did not exhibit significant statistical differences.

**Table 1:** Overall Demographic and Clinical Information Statistics for the ROP Cohort and TR-ROP Cohort in SZEH i-ROP Dataset

Characteristics	Overall	ROP Cohort			TR-ROP Cohort		
		ROP *n* = 3335 (14.8%)	no-ROP *n* = 19,234 (85.2%)	*p*	TR-ROP *n* = 1234 (5.5%)	no-TR-ROP *n* = 21,335 (94.5%)	*p*
BW, g, mean (95%CI; SD)	1480.5 (1476.1–1484.9; 334.3)	1203.2 (1192.6–1213.8; 311.4)	1528.6 (1524.1–1533.0; 314.2)	< 0.001^b^	1150.5 (1133.5–1167.6; 305.2)	1499.6 (1495.2- 1504.0; 325.9)	< 0.001 ^b^
GA, weeks, mean (95%CI; SD)	31.4 (31.4–31.5; 2.4)	29.1 (29.1–29.2; 2.1)	31.8 (31.8–31.9; 2.2)	< 0.001 ^b^	28.6 (28.5–28.7; 2.1)	31.6 (31.6–31.6; 2.3)	< 0.001 ^b^
No. of women (%)	9915 (43.9)	1278 (38.3)	8637 (44.9)	< 0.001^a^	447 (36.3)	9468 (44.4)	< 0.001^a^
No. MB (%)	6921 (30.7)	994 (29.8)	5927 (30.8)	0.243 ^a^	376 (30.5)	6545 (30.7)	0.878 ^a^
No. CS (%)	13,928 (61.7)	1562 (46.8)	12,366 (64.3)	< 0.001 ^a^	476 (38.6)	13,452 (63.1)	< 0.001 ^a^

*BW* Birth weight, *GA* Gestational age, *MB* multiple births, *CS* Caesarean section, *SD* Standard deviations, *CI* confidence interval; ^a^ By Chi-Squared test; ^b^ By Mann-Whitney U test.

Subsequently, based on the four risk factors identified with significant statistical differences in the single-factor analysis, a binary logistic regression model was employed to conduct a multi-factor analysis for both ROP and TR-ROP risk factors. The results of the multi-factor analysis were presented in Table 2. After analysis, we finally identified four factors, including BW (*p* < 0.001, p < 0.001), GA (*p* < 0.001, *p* < 0.001), gender (*p* < 0.001, *p* < 0.001), and MD (*p* = 0.008, *p* < 0.001), as predictive risk factors for ROP and TR-ROP.

**Table 2:** Multi-factor analysis results for ROP and TR-ROP risk prediction

Risk factor	Total	ROP Cohort				TR-ROP Cohort			
		ROP	no-ROP	OR (95%CI)	*p*	TR-ROP	no-TR-ROP	OR (95%CI)	*p*
BW	22,569	3335 (14.8%)	19,234 (85.2%)	0.999 (0.999–0.999)	< 0.001	1234 (5.5%)	21,335 (94.5%)	0.999 (0.999–0.999)	< 0.001
GA	22,569	3335 (14.8%)	19,234 (85.2%)	0.664 (0.646–0.683)	< 0.001	1234 (5.5%)	21,335 (94.5%)	0.648 (0.619–0.677)	< 0.001
Gender					< 0.001				< 0.001
Man	12,654	2057 (16.3%)	10,597 (83.7%)	1.00		787 (6.2%)	11,867 (93.8%)	1.00	
Women	9915	1278 (12.9%)	8637 (87.1%)	0.808 (0.742–0.881)		447 (4.5%)	9468 (95.5%)	0.772 (0.679–0.878)	
MD					0.008				< 0.001
CS	13,928	1562 (11.2%)	12,366 (88.8)	1.00		476 (3.4%)	13,452 (96.6%)	1.00	
Non-CS	8641	1773 (20.5%)	6868 (79.5%)	1.124 (1.031–1.225)		758 (8.8%)	7883 (91.2%)	1.389 (1.220–1.582)	

*BW* Birth weight, *GA* Gestational age, *MD* mode of delivery, *CS* Caesarean section, *CI* confidence interval.

Finally, these 22,569 patients were evenly divided into five groups based on their final diagnostic results respectively, and these five groups were used in subsequent five-fold cross-validation experiments. The patients in the training and testing datasets were mutually exclusive.

### Risk model development

From the single-factor analysis and multi-factor analysis, we can observe that BW, GA, Gender, and MD may be the major factors in risk prediction of ROP and TR-ROP. In this study, two machine learning methods of logistic regression and decision tree and a deep learning method of multilayer perceptron (MLP) were used to achieve ROP and TR-ROP risk prediction. All three models utilized a five-fold cross-validation strategy for model optimization. Logistic regression incorporated L2 regularization as a penalty term and lbfgs as the optimization algorithm. Decision tree utilized entropy based on Shannon information gain to measure the quality of a split, employing a random strategy for selecting the best random split at each node. In MLP, five linear layers were designed, with the output of the first four linear layers sequentially processed through a ReLU activation layer and a batch normalization layer. The last linear layer was used for the final classification.

### Model evaluation

The dataset used in this study had class imbalance, with the proportion of preterm infants with ROP being 14.8% compared to no-ROP. Among ROP patients, TR-ROP accounts for 37%. Therefore, we mainly chose the area under the precision-recall curve (AUCPR) and the area under the receiver operating characteristic curve (AUC) as the main measures of model performance. DeLong’s test was used to compare the AUCs of different models. Additionally, evaluation metrics such as accuracy (Acc), sensitivity (Sen) and specificity (Spec) are included. Acc calculates the ratio of the number of correctly predicted samples (true positives and true negatives) to the total number of samples (true positives, false negatives, false positives, and true negatives). Sen measures the proportion of true positive samples predicted by the model among all actual positive samples. Spec measures the proportion of true negative samples predicted by the model among all actual negative samples. To calculate these metrics, we utilize the following formulas:

$$\textrm{Acc}:\left(\textrm{TP}+\textrm{TN}\right)/\left(\textrm{TP}+\textrm{FN}+\textrm{FP}+\textrm{TN}\right)$$

$$\textrm{Sen}:\textrm{TP}/\left(\textrm{TP}+\textrm{FN}\right)$$

$$\textrm{Spec}:\textrm{TN}/\left(\textrm{TN}+\textrm{FP}\right)$$

TP, TN, FP, FN and TN represent true positives, true negatives, false positives, false negatives, true negatives respectively.

## Results

### Data characteristics

**Fig. 1:**
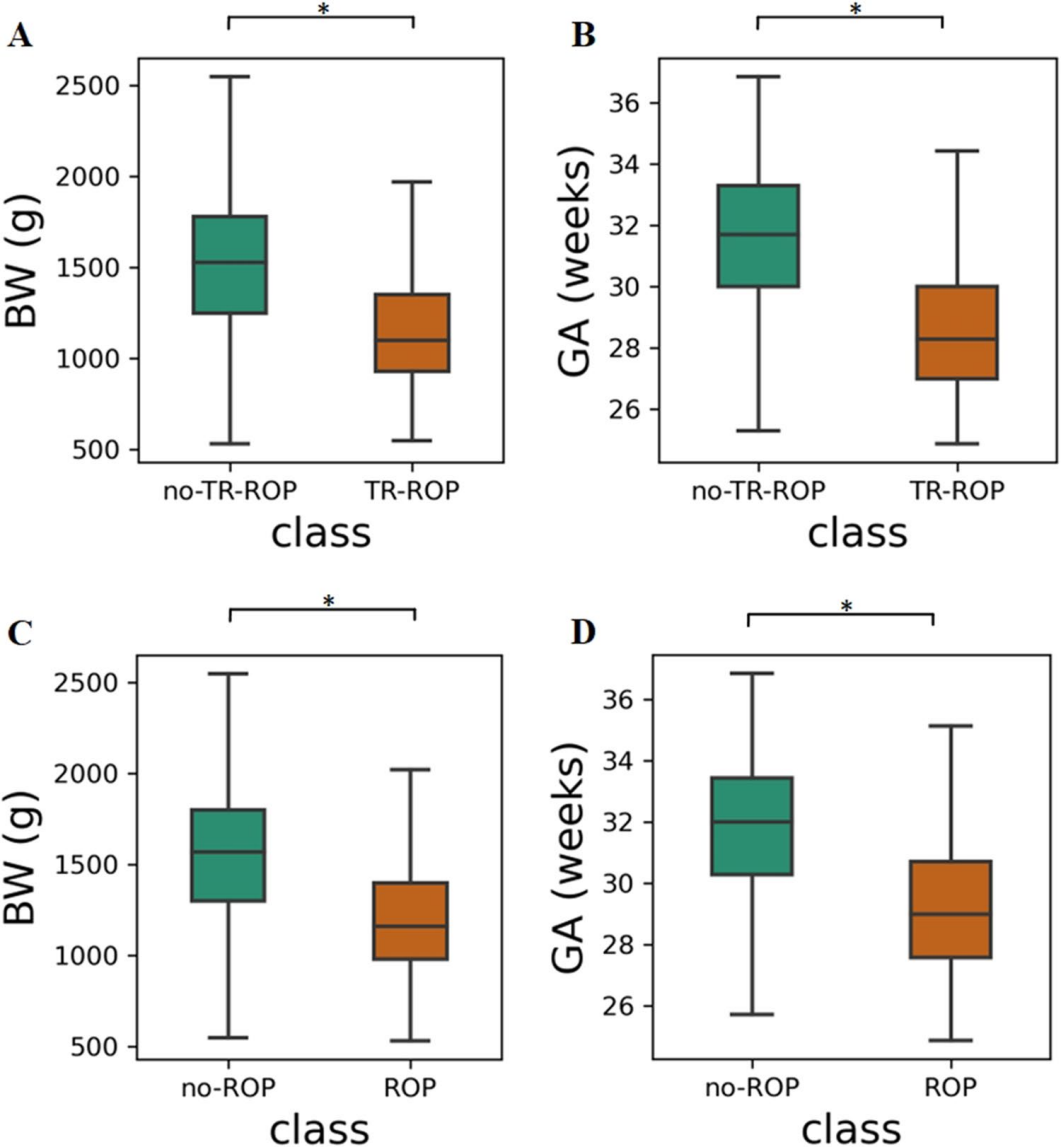
Box plot of BW and GA in TR-ROP Cohort (**A**, **B**) and ROP Cohort (**C**, **D**). There is a significant difference in BW (*p* < 0.001) and GA (*p* < 0.001) between no-ROP and ROP. There is also a significant difference in BW (*p* < 0.001) and GA (*p* < 0.001) between no-TR-ROP and TR-ROP. * *p* < 0.05

This study used demographic data and clinical outcomes from 22,569 infants enrolled in the SZEH between 2003 and 2023. Tables 1 and 2 displays relevant demographic data and clinical outcomes from the dataset. Among those infants, 9915 were female (43.9%). The mean (SD) GA was 31.4 (2.4) weeks, and the mean (SD) BW was 1480.5 (334.3 g). Among those infants, 3335 were diagnosed with ROP, with 1234 infants identified as TR-ROP. The median (interquartile range, IQR) BW for no-ROP infants is 1570 g (1300.0–1800.0 g), while for infants with ROP, it is 1160 g (980.0–1400.0 g). The median (IQR) GA for no-ROP infants is 32.0 weeks (30.3–33.4 weeks), whereas for ROP infants, it is 29.0 weeks (27.6–30.7 weeks). The median (IQR) BW for no-TR-ROP infants was 1499.6 g (1495.2–1504.0 g), while for TR-ROP infants, it was 1150.5 g (1133.5–1167.6 g). The median (IQR) GA for no-TR-ROP infants was 31.6 weeks (31.569–31.631 weeks), whereas for TR-ROP infants, it was 28.6 weeks (28.5–28.7 weeks). Figure 1 illustrated the distribution of infants in the dataset based on BW and GA.

### Performance in ROP risk prediction models

We employed two machine learning methods and one deep learning method to predict the risk of ROP. From the multi-factor analysis results of ROP risk prediction in Table 2, it was evident that BW (*p* < 0.001), GA (*p* < 0.001), gender (*p* < 0.001), and MD (*p* = 0.008) were the primary predictive risk factors for ROP risk. We designed four different combinations of risk factors, and all three models explored the optimal combination of risk factors within these four combinations.

**Fig. 2:**
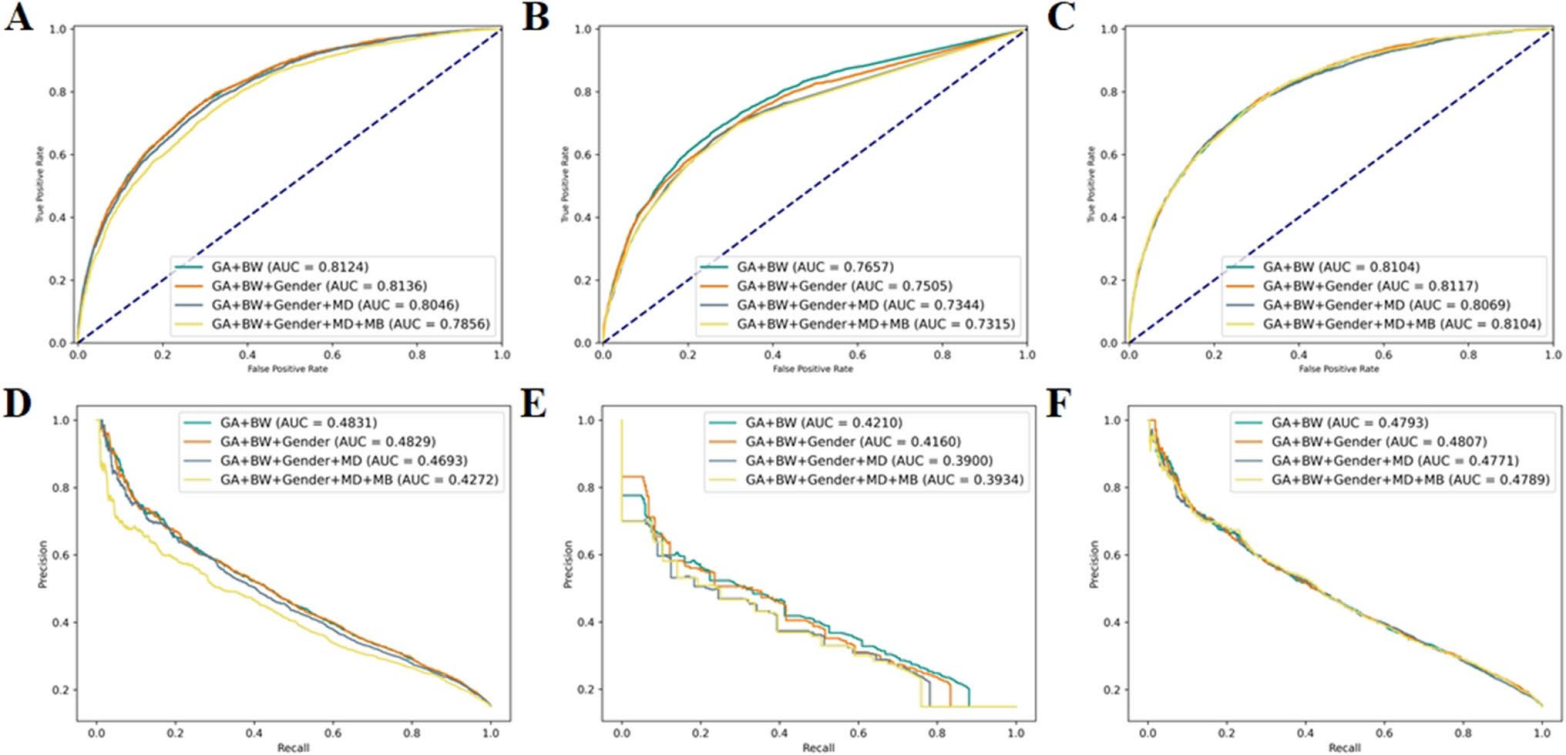
The comprehensive ROC curve and PR curve for each model’s five-fold cross-validation results in ROP risk prediction. **A**, ROC curve for logistic regression. **B**, ROC curve for decision tree. **C**, ROC curve for MLP. **D**, PR curve for logistic regression. **E**, PR curve for decision tree. **F**, PR curve for MLP

**Fig. 3:**
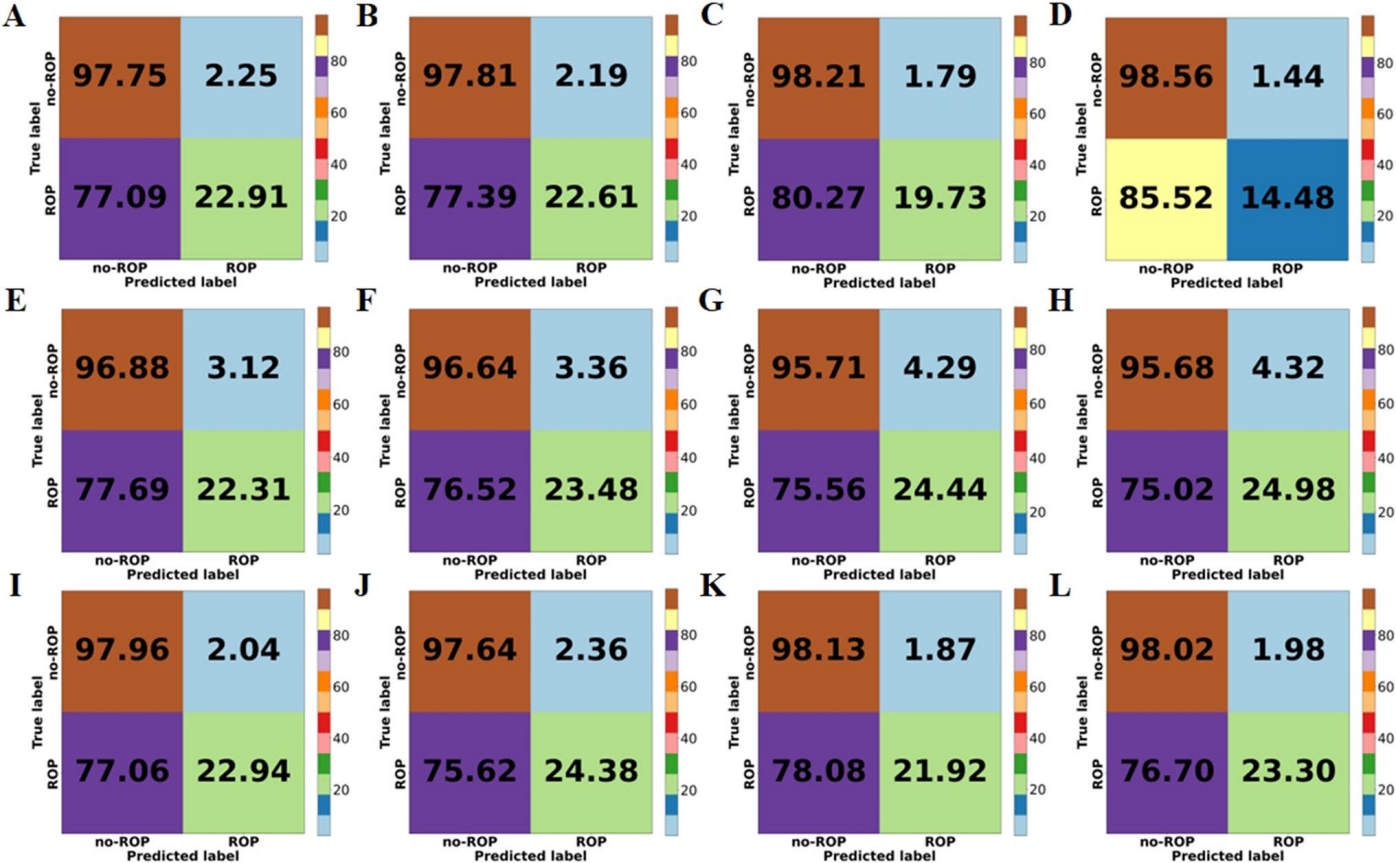
The comprehensive confusion matrix for each model’s five-fold cross-validation results in ROP risk prediction. **A**: GA + BW, **B**: GA + BW + Gender, **C**: GA + BW + Gender + MD, **D**: GA + BW + Gender + MD + MB, for logistic regression. **E**: GA + BW, **F**: GA + BW + Gender, **G**: GA + BW + Gender + MD, **H**: GA + BW + Gender + MD + MB, for decision tree. **I**: GA + BW, **J**: GA + BW + Gender, **K**: GA + BW + Gender + MD, **L**: GA + BW + Gender + MD + MB, for MLP

The experimental results were shown in Table 3. In the five-fold cross-validation of logistic regression, the combination GA + BW performed the best among the three metrics: AUCPR, Acc, and Sen. Although it did not achieve the highest AUC compared to the combination GA + BW + Gender (81.36 (80.90–81.82)), the difference was only 0.12%. DeLong’s test showed no significant difference in AUC between them (*p* = 0.973). Except for the Spec, the comprehensive performance of the combination GA + BW + Gender + MD + MB was the worst (GA + BW vs GA + BW + Gender + MD + MB, *p* < 0.001). The combination GA + BW + Gender + MD had an AUC 0.83% lower than GA + BW, although it did not show a significant difference (*p* = 0.072). In decision tree, the combination GA + BW achieved the best performance in all four metrics, and AUC showed a significant difference compared to the other three combinations (GA + BW vs GA + BW+ Gender, *p* = 0.021; GA + BW vs GA + BW + Gender + MD, *p* < 0.001; GA + BW vs GA + BW + Gender + MD + MB, *p* < 0.001). In the results of the four combinations in MLP, the AUC were relatively close, with no significant differences observed (GA + BW vs GA + BW+ Gender, *p* = 0.958; GA + BW vs GA + BW + Gender + MD, *p* = 0.329; GA + BW vs GA + BW + Gender + MD + MB, *p* = 0.828). According to the comprehensive results of the three models, the combination GA + BW had certain advantages in the risk prediction of ROP, and the logistic regression had the best effect among the three methods. Although in the DeLong’s test results for AUC under the GA + BW combination, MLP did not show significant differences (logistic regression vs decision tree, *p* < 0.001; logistic regression vs MLP, *p* = 0.5413). The comparative ROC and PR curves and confusion matrix for the three models were shown in Figs. 2 and 3.

**Table 3:** Five-fold cross-validation results of each combination of risk factors for ROP risk prediction using 3 models

Method	Combinations	AUC^a^	AUCPR^a^	Acc^a^	Sen^a^	Spec^a^	p^*^
logistic regression	GA + BW	81.24 (80.83–81.65)	**48.49** (46.32–50.66)	**86.69** (86.23–87.15)	**22.91** (22.10–23.72)	97.75 (97.10–98.40)	–
	GA + BW+ Gender	**81.36** (80.90–81.82)	48.44 (46.22–50.66)	86.69 (86.19–87.19)	22.61 (21.27–23.95)	97.81 (97.21–98.41)	0.973
	GA + BW + Gender + MD	80.41 (77.95–82.87)	47.10 (42.41–51.79)	86.61 (86.05–87.17)	19.73 (12.18–27.28)	98.21 (97.27–99.15)	0.072
	GA + BW + Gender + MD + MB	78.53 (75.87–81.19)	42.84 (37.25–48.43)	86.14 (85.48–86.80)	14.48 (5.83–23.13)	**98.56** (97.53–99.59)	< 0.001
decision tree	GA + BW	**76.57** (75.89–77.25)	**42.21** (40.47–43.95)	**85.86** (85.46–86.26)	22.31 (18.66–25.96)	**96.88** (96.23–97.53)	–
	GA + BW+ Gender	75.08 (74.32–75.84)	41.70 (39.80–43.60)	85.83 (85.46–86.20)	23.48 (22.44–24.52)	96.64 (96.12–97.16)	0.021
	GA + BW + Gender + MD	73.43 (72.69–74.17)	39.16 (37.67–40.65)	85.18 (84.62–85.74)	24.44 (22.99–25.89)	95.71 (95.21–96.21)	< 0.001
	GA + BW + Gender + MD + MB	73.16 (72.33–73.99)	39.38 (38.55–40.21)	85.24 (85.12–85.36)	**24.98** (23.07–26.89)	95.68 (95.3–96.06)	< 0.001
MLP	GA + BW	81.23 (80.73–81.73)	**48.43** (46.23–50.63)	86.88 (86.41–87.35)	22.94 (18.20–27.68)	97.96 (97.34–98.58)	–
	GA + BW+ Gender	**81.29** (80.76–81.82)	48.40 (46.18–50.62)	86.82 (86.34–87.3)	**24.38** (19.61–29.15)	97.64 (97.06–98.22)	0.958
	GA + BW + Gender + MD	81.21 (80.85–81.57)	48.38 (46.33–50.43)	86.87 (86.40–87.34)	21.92 (16.62–27.22)	**98.13** (97.43–98.83)	0.329
	GA + BW + Gender + MD + MB	81.12 (80.52–81.72)	48.40 (46.43–50.37)	**86.98** (86.63–87.33)	23.30 (18.41–28.19)	98.02 (97.19–98.85)	0.828

*BW* Birth weight, *GA* Gestational age, *MB* Multiple births, *MD* Mode of delivery, *CS* Caesarean section; ^a^Mean ± SD results from five-fold cross-validation; *Comparison of AUC with that of different risk factor combinations by using DeLong’s test.; the contents of () represent 95% CI.

### Performance in TR-ROP risk prediction models

**Fig. 4:**
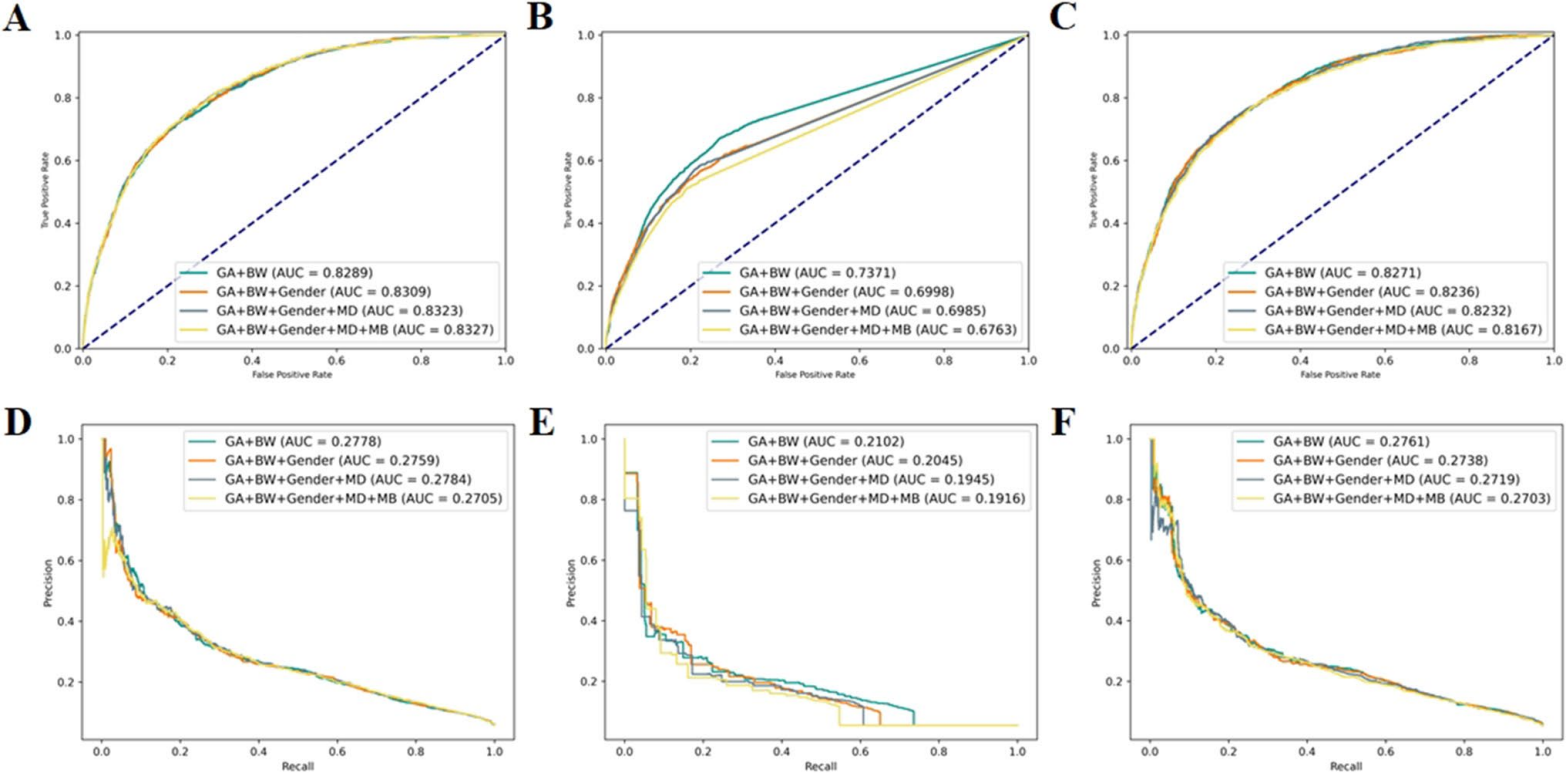
The comprehensive ROC curve and PR curve for each model’s five-fold cross-validation results in TR-ROP risk prediction. **A**, ROC curve for logistic regression. **B**, ROC curve for decision tree. **C**, ROC curve for MLP. **D**, PR curve for logistic regression. **E**, PR curve for decision tree. **F**, PR curve for MLP

The results of the three TR-ROP risk prediction models were presented in Table 4. In logistic regression, the combination GA + BW + Gender + MD + MB demonstrated the best overall performance, with the highest AUC of 83.28 (82.19–84.37). With this combination of risk factors, the diagnostic Sen reached 75.36% and the Spec reached 74.83%, which meant that the logistic regression was able to identify about 75% of infants who need treatment and nearly 75% of infants who did not need treatment. Although there were no significant differences compared to other combinations (GA + BW + Gender + MD + MB vs GA + BW, *p* = 0.601; GA + BW + Gender + MD + MB vs GA + BW+ Gender, *p* = 0.760; GA + BW + Gender + MD + MB vs GA + BW + Gender + MD, *p* = 0.948). In decision tree, the combination GA + BW performed best in overall performance, showing significant differences in AUC compared to other combinations (*p* < 0.001 for all). However, its highest AUC is only 73.68 (71.66–75.70). In MLP, the combination GA + BW achieved the best AUC and AUCPR, but there were no significant differences compared to other combinations (GA + BW vs GA + BW+ Gender, *p* = 0.485; GA + BW vs GA + BW + Gender + MD, *p* = 0.215; GA + BW vs GA + BW + Gender + MD + MB, *p* = 0.101). Among the three models, logistic regression had the highest AUC and AUCPR in the combination GA + BW + Gender + MD + MB. Compared to the best combinations in the other two models, DeLong’s test results showed significant differences in decision tree (*p* < 0.001) and no significant differences in MLP (*p* = 0.348). The comparative ROC and PR curves for the three models were shown in Fig. 4.

**Table 4:** Five-fold cross-validation results of each combination of risk factors for TR-ROP risk prediction using 3 models

Method	Combinations	AUC^a^	AUCPR^a^	Acc^a^	Sen^a^	Spec^a^	p^*^
logistic regression	GA + BW	82.91 (81.61–84.21)	**27.89** (24.74–31.04)	74.02 (73.26–74.78)	74.63 (71.53–77.73)	73.99 (73.05–74.93)	0.601
	GA + BW+ Gender	83.12 (81.92–84.32)	27.67 (24.68–30.66)	74.38 (73.75–75.01)	75.20 (72.06–78.34)	74.33 (73.54–75.12)	0.760
	GA + BW + Gender + MD	83.25 (82.03–84.47)	27.87 (24.77–30.97)	74.81 (74.28–75.34)	**75.68** (73.28–78.08)	74.76 (74.11–75.41)	0.948
	GA + BW + Gender + MD + MB	**83.28** (82.19–84.37)	27.13 (24.47–29.79)	**74.86** (74.33–75.39)	75.36 (71.98–78.74)	**74.83** (74.16–75.50)	–
decision tree	GA + BW	**73.68** (71.66–75.70)	**21.07** (18.85–23.29)	94.36 (94.24–94.48)	5.51 (3.60–7.42)	**99.50** (99.36–99.64)	–
	GA + BW+ Gender	69.97 (69.00–70.94)	20.62 (18.06–23.18)	**94.47** (94.42–94.52)	6.81 (5.68–7.94)	99.54 (99.40–99.68)	< 0.001
	GA + BW + Gender + MD	69.88 (68.49–71.27)	19.67 (17.62–21.72)	94.16 (93.99–94.33)	8.75 (6.73–10.77)	99.10 (98.89–99.31)	< 0.001
	GA + BW + Gender + MD + MB	67.65 (65.91–69.39)	19.25 (16.93–21.57)	94.19 (94.02–94.36)	**9.15** (6.44–11.86)	99.11 (98.94–99.28)	< 0.001
MLP	GA + BW	**82.84** (81.69–83.99)	**27.93** (24.38–31.48)	94.75 (94.69–94.81)	5.67 (4.02–7.32)	99.91 (99.84–99.98)	–
	GA + BW+ Gender	82.61 (81.53–83.69)	27.76 (24.79–30.73)	94.74 (94.67–94.81)	5.11 (2.66–7.56)	**99.92** (99.82–100.00)	0.485
	GA + BW + Gender + MD	82.50 (80.82–84.18)	27.80 (24.83–30.77)	**94.77** (94.72–94.82)	**7.13** (6.20–8.06)	99.84 (99.78–99.90)	0.215
	GA + BW + Gender + MD + MB	82.08 (80.80–83.36)	27.10 (23.64–30.56)	94.74 (94.65–94.83)	6.23 (4.40–8.06)	99.86 (99.82–99.9)	0.101

*BW* Birth weight, *GA* Gestational age, *MB* multiple births, *MD* mode of delivery, *CS* Caesarean section; ^a^Mean ± SD results from five-fold cross-validation; *Comparison of AUC with that of different risk factor combinations by using DeLong’s test; the contents of () represent 95% CI.

## Discussion

### Predictive approach

Early detection, prevention, and intervention of ROP are key to reducing incidence and mortality and alleviating the huge socio-economic burden caused by ROP. Screening out the factors with significant statistical differences from numerous risk factors and exploring the best combination of risk factors are an effective strategy for predictive diagnostic, targeted prevention and personalized treatment of ROP [13, 29]. Low-risk preterm infants should receive fewer examinations to alleviate scarce medical resources, while high-risk infants (premature infants with ROP who require treatment) should be treated with medication or laser surgical interventions to reduce the risk of blindness, which effectively promotes the paradigm shift from reactive medicine to the advanced approach by utilizing the 3 PM framework. The active promotion of the PPPM framework has been applied in many fields, making great contributions to the health quality improvement including fundus diseases [32], diabetes [33], cancers [34], etc. In this study, we retrospectively evaluated an AI system for ROP risk and TP-ROP risk prediction developed for preterm infants in Chinese population based on the i-ROP cohort study dataset from SZEH. First, statistical single-factor analysis was used to determine the risk factors with statistically significant differences among the five risk factors for ROP and TR-ROP. Then, based on the risk factors with significant statistical differences found in the single-factor analysis, a binary logistic regression model was used for multi-factor analysis to determine the final risk prediction factors. Finally, with statistically significant risk factors as input, two machine learning methods of logistic regression and decision tree and a deep learning method of MLP were used to explore the optimal prediction method for ROP and TR-ROP risk prediction.

**Fig. 5:**
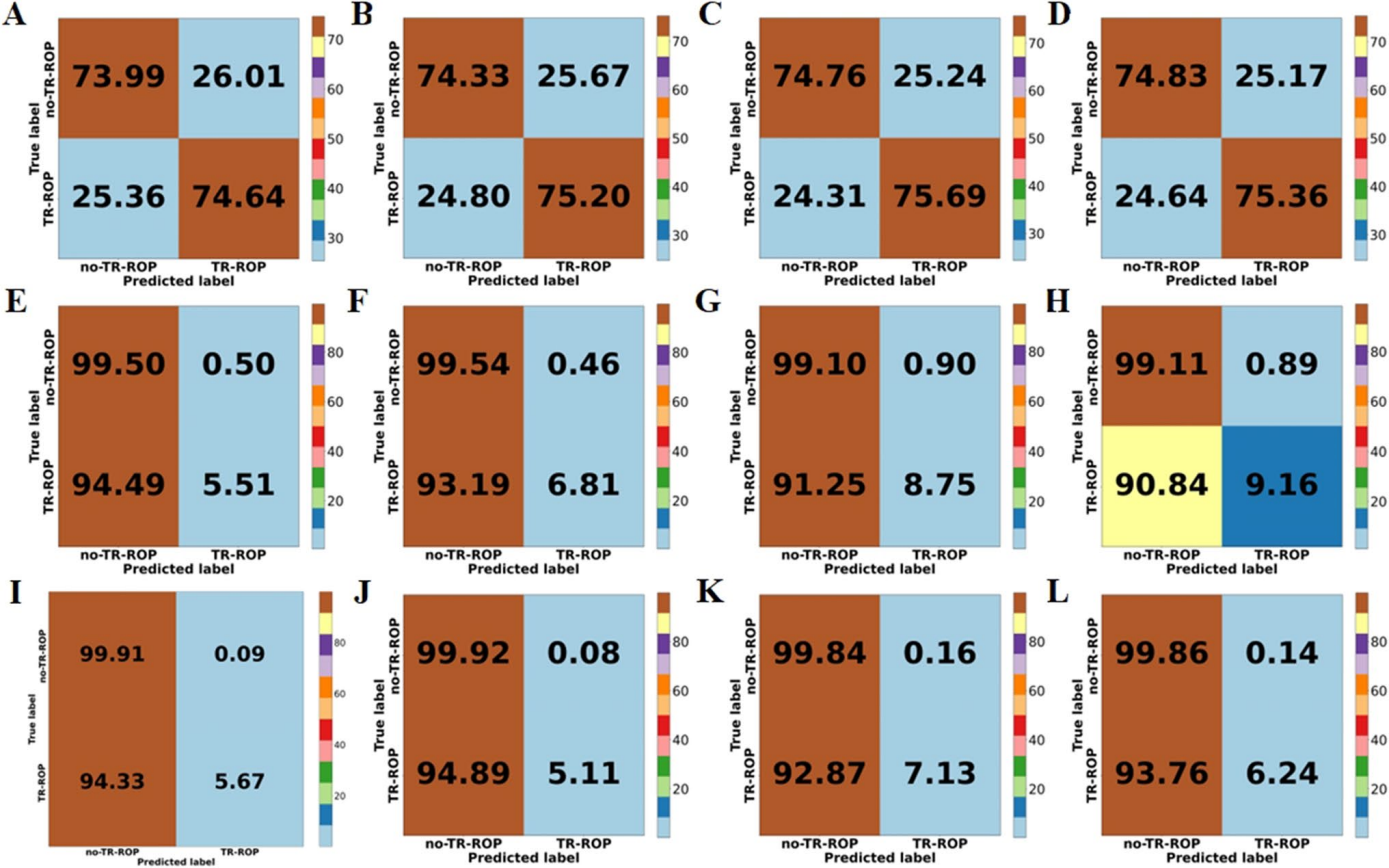
The comprehensive confusion matrix for each model’s five-fold cross-validation results in TR-ROP risk prediction. **A**: GA + BW, **B**: GA + BW + Gender, **C**: GA + BW + Gender + MD, **D**: GA + BW + Gender + MD + MB, for logistic regression. **E**: GA + BW, **F**: GA + BW + Gender, **G**: GA + BW + Gender + MD, **H**: GA + BW + Gender + MD + MB, for decision tree. **I**: GA + BW, **J**: GA + BW + Gender, **K**: GA + BW + Gender + MD, **L**: GA + BW + Gender + MD + MB, for MLP

We observed that among the three AI-based ROP risk prediction models, logistic regression has better comprehensive performance. Only using BW and GA can identify approximately 23% of infants who are at risk of ROP before being diagnosed with ROP, while simultaneously excluding 97.75% of those at low risk (Table 3, Fig. 3-A). When other risk factors were added to the combination of GA + BW, both logistic regression and MLP showed improvement in specificity, which indicated that more low-risk people could be excluded. In MLP, adding gender can achieve better sensitivity compared to the combination GA + BW (Fig. 3). In addition, we knew that in the results of single-factor analysis and multi-factor analysis for risk factors (Tables 1 and 2), MD is a risk factor with significant statistical difference, but there is no significant improvement after adding MD to the ROP risk prediction methods. Instead, a certain performance decline was observed in all three methods (AUC and AUCPR exhibited a decrease in Fig. 2). On the other hand, although there is no statistically significant difference in the risk factor MB, the model’s specificity was improved by adding MB to logistic regression (Fig. 3). Among the three AI-based TR-ROP risk prediction models, logistic regression demonstrated relatively superior performance. Using the risk factor combination GA + BW + Gender + MD + MB, 75.36% of children who need treatment could be identified, while excluding nearly 75% of infants who did not require treatment (Table 3, Fig. 5-D). Moreover, we found that there was no significant difference in AUC between the two methods of logistic regression and MLP in the four risk factor combinations (Table 4).

**Fig. 6:**
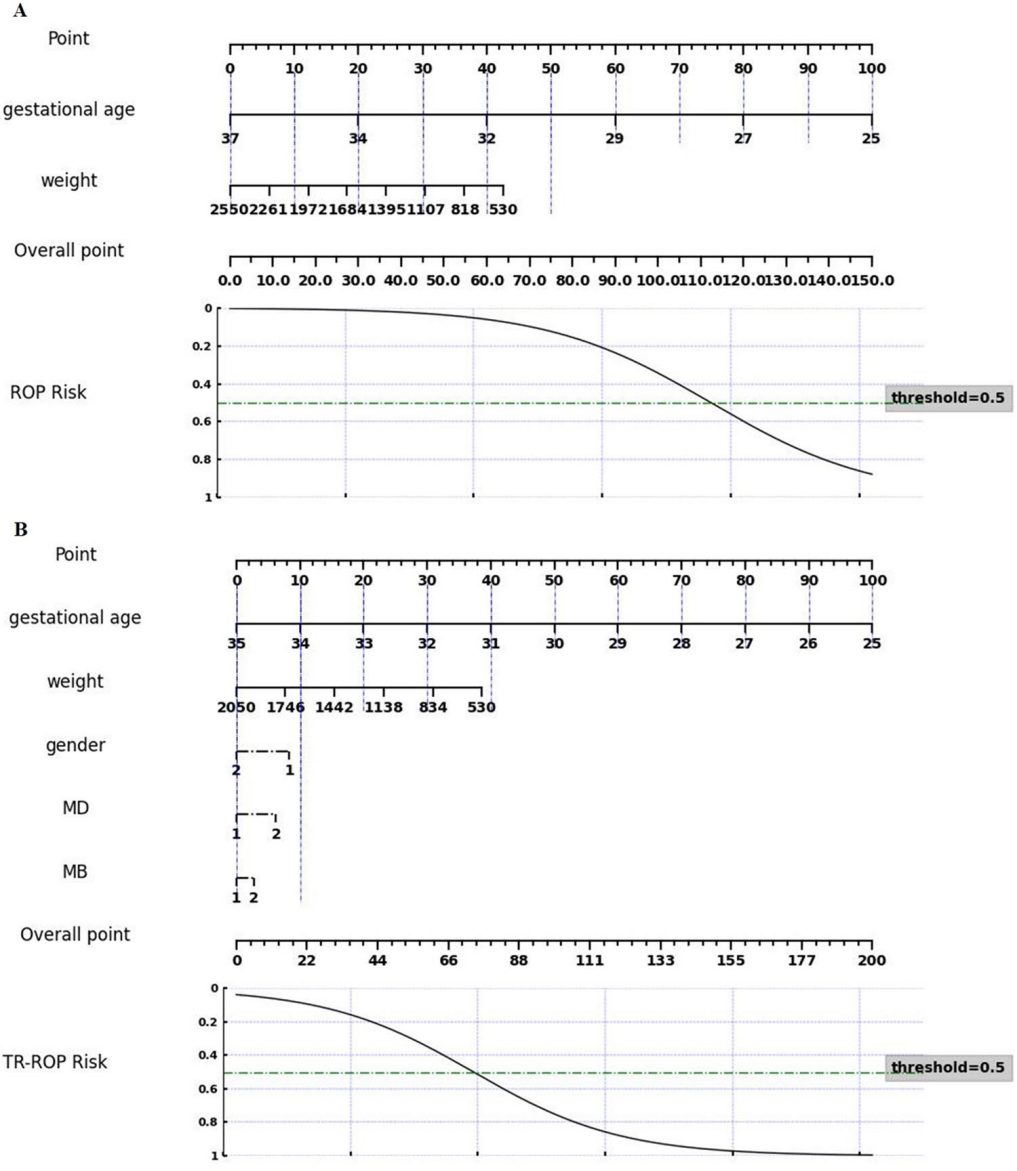
Nomograms of prevention models. **A**: Nomogram of ROP risk prediction, **B**: Nomogram of TR-ROP risk prediction

It can be seen from the result analysis that the comprehensive performance of logistic regression is better, BW + GA risk factor combination can obtain the best performance in ROP risk prediction, and the risk factor combination GA + BW+ Gender + MD + MB has the best performance in TR-ROP risk prediction. We established a new ROP and TP-ROP prediction model based on the combination of these risk factors. The formula of ROP risk prediction model was as follows:

1 $$\textrm{Risk}\ \textrm{of}\ \textrm{ROP}=\frac{1}{1+{\textrm{e}}^{12.994-0.4344{x}_1-0.0011{x}_2}}$$

The formula of TR-ROP prediction model is as follows:

2 $$\textrm{Risk}\ \textrm{of}\ \textrm{TR}-\textrm{ROP}=\frac{1}{1+{\textrm{e}}^{14.5123-0.4331{x}_1-0.0012{x}_2-0.3617{x}_3+0.2714{x}_4+0.1217{x}_5}}$$

Where x_1_ represents GA, x_2_ represents BW, x_3_ represents gender, x_4_ represents MD, and x_5_ represents MB. In the context of PPPM [35], efforts to incorporate this risk prediction paradigm into current clinical workflows would be advantageous, as it has the potential to become a promising tool for early detection and treatment of ROP disorders in infants.

### Targeted prevention

In terms of prevention [35], ROP was one of the main causes of blindness in children, and early detection can help prevent further deterioration of the condition in time and stop the progression of the disease. Our findings indicated that using the five easily available ROP risk factors for early ROP risk and TR-ROP risk prediction can help reduce the burden on ophthalmologists and improve screening efficiency, especially in low and middle-income regions. In this study, for a targeted ROP prevention model designed for a specific Chinese population, we further simplified the model into nomograms as shown in Fig. 6, so that it can be more convenient to enter the clinical application.

The above situation was obtained from the analysis of a specific Chinese population, and the model is unlikely to generalize well “out of the box” to populations different from the Chinese screening population. Depending on epidemiological and demographic risk factors, the prediction models need to be re-adjusted based on local disease epidemiology, and different risk factor combinations may have different performance because the incidence of ROP varies geographically and over time [29, 30]. We believed that these results prove that the AI-based risk prediction model can effectively reduce the number of ROP examinations and associated physiological stress in low-risk infants, thereby improving the efficiency of ROP screening, and can also be used as an epidemiological tool to monitor NICU-level ROP risk and TP-ROP risk prediction across regions and time. Finally, quantitative monitoring that combines GA and BW, the two strongest risk factors for developing ROP, with other risk factors could allow for earlier and more consistent diagnosis of TR-ROP, thereby minimizing the overall risk of adverse outcomes.

This model may also be easier to implement than the existing ROP risk prediction models. Coyner et al. [13] proposed a method for TR-ROP risk prediction using a combination of GA and Vascular Severity Score (VSS) factors and achieved good performance. However, in this method, an additional AI model needs to be trained to obtain the VSS based on the patient’s fundus images, which is relatively cumbersome in clinical practice because the images are not part of the standard of care and digital fundus cameras may be expensive, so in some low- or middle-income countries or regions, fundus images of infants are not easily available. Moreover, the best AUC obtained by this method was 0.82, while our prediction model could reach 0.83. The specificity of our proposed risk prediction model was much higher than that of the ROP prediction model of the Children’s Hospital of Philadelphia [36], which uses BW + GA + weight gain to predict the risk of type II ROP and TR-ROP, and the highest specificity was 53.4%, while in in our TR-ROP prediction model, the specificity reached 74.83%.

### Personalization of medical services

There are many risk factors for ROP [29], and this study was based on the analysis of a specific Chinese population. In practice, when the model was extended to a population different from the Chinese screening population, it was recommended to appropriately adjust the input parameters of the model according to the local disease epidemiology. Different combinations of risk factors may have different impacts on the performance of the risk prediction model. This paradigm of ROP and TR-ROP risk prediction by using ROP risk factors as input to the model will enable high-risk infants receive earlier and more accurate diagnosis and treatment, while minimizing the over-examination of low-risk infants, and better reduce or eliminate the occurrence of ROP-related blindness. In this study, the overall sensitivity of the ROP risk prediction model was relatively low, and it may be necessary to continue to integrate oxygen exposure, intraventricular hemorrhage, neonatal sepsis, necrotizing enterocolitis, thrombocytopenia, and other previously associated risk factors [29] to further improve the model’s sensitivity.

For the automatic identification of ROP, many deep learning (DL) algorithms were currently developed based on color fundus photography images. For example, Brown et al. [23] used two CNNs to realize the automatic diagnosis of plus disease in ROP. First, a U-Net architecture was used to segment blood vessels, and then an Inception network [37] was used to realize the classification task of plus disease in ROP. Furthermore, there were studies using DL algorithms to achieve quantitative assessment of ROP risk severity. For example, Taylor et al. [24] used a DL algorithm for vascular severity scoring to identify TR-ROP by quantifying clinical disease progression, while Kellyn et al. [38] used a vascular severity score based on DL to describe aggressive posterior retinopathy of prematurity quantitatively. In addition, there are also some studies to implement automatic classification tasks of ROP. For example, Zhang et al. [39] used a VGG-16 network to realize the automatic classification task of ROP. Wang et al. [40] developed an automatic ROP detection system using two deep neural networks, in which the Id-Net network was used to identify the ROP, the Gr-Net network was further used to grade the severity of ROP. With the popularity of smartphones, smartphone-based cameras may become a substitute for professional color fundus photography, and the acquisition of infant fundus images may become more convenient. According to fundus images, more clinical characteristics can be obtained, such as the development of blood vessels in different fundus zones. Whether combining that information with the factors previously involved can further improve the prediction performance of the model is worth exploring. If possible, this would not only greatly reduce the burden of ROP screening, but also enable high-risk infants to receive timely treatment and reduce the burden on society. In any case, the risk prediction model proposed in this study can lay a basic framework for a new ROP prediction model, which can at least achieve ROP risk prediction and TR-ROP risk prediction only by inputting GA and BW, without requiring a complete ophthalmoscopy. This paradigm allows low-risk infants to receive fewer examinations, while high-risk infants receive earlier, more precise diagnosis and treatment. This could allow rural areas and low- and middle-income countries to make better use of scarce resources.

## Conclusion, outlook in the framework of PPPM/3 PM, and limitations of the study

### Conclusion

Our study found that birth weight, gestational age, multiple births; mode of delivery, gender were related to the occurrence of ROP and TR-ROP in specific Chinese populations. We employed multiple risk factors to explore predictive models for ROP risk and TR-ROP risk, identifying the best model through rich comparative experiments of three different models. In the SZEH’s i-ROP cohort study dataset, we demonstrated that using only the two main risk factors of BW and GA not only could identify approximately 23% of infants at risk for ROP before ROP is diagnosed, but excluded 97.75% of low-risk infants. It can also identify about 75% of infants who need treatment and nearly 75% of children who did not need treatment were detected. We also constructed a simple and easy to operate nomogram model for clinical staff to use. The implementation of this model can significantly reduce the number of ROP examinations in low-risk infants, and can identify TR-ROP infants at an early stage and give timely treatment, so as to better utilize ROP screening resources. Future work will verify the effectiveness of this paradigm in a larger population and explore whether using more relevant risk factors can further improve the predictive performance of the model and better reduce or eliminate the occurrence of blindness due to ROP.

### Limitations and outlook in the framework of PPPM/3 PM

This study has several limitations. Our i-ROP cohort study dataset is relatively small, contained only Chinese individuals, and was limited to a single geographic region, limiting its generalizability. Therefore, in future work, databases from different regions and other parts of the world should be expanded to increase the data diversity and improve the applicability of risk prediction models. The ROP risk prediction model designed in this study is a non-invasive automated system for early prediction of ROP and TR-ROP in infants. It also aligns with a key aspect of the PPPM/3 PM approach highlighted at the 2019 EPMA World Congress, which focuses on customizing and continuously monitoring patients’ clinical parameters to improve treatment outcomes [41]. Additionally, this study considered only five factors associated with ROP risk, and the overall sensitivity of the ROP disease risk prediction model is relatively low. In the future, incorporating more risk factors for analysis should be considered to enhance the sensitivity of the prediction model. To facilitate future applications of AI in this field, it is strongly recommended to consider the following two points:

1. Ensure that biological information about ROP is accurately recorded at birth, including but not limited to risk factors such as BW, GA, gender, MD, and MB, so as to provide real and effective data support for the subsequent disease risk prediction models.
2. In order to be able to predict disease progression comprehensively, it is recommended to register follow-up records in a timely manner to facilitate the evaluation of treatment decisions relevant to the clinical setting, so that predictive models can be applied to clinical risk monitoring at an early stage. Guided by the PPPM/3 PM approach, better clinical outcomes for patients are provided by providing complete prediction from diagnosis to treatment decisions.

## Data Availability

No datasets were generated or analysed during the current study.

## Code availability

The source code used in this study is available upon request from the first author only.

## Abbreviations

ROP: Retinopathy of prematurity
TR-ROP: Treatment-requiring retinopathy of prematurity
AI: Artificial intelligence
i-ROP: Informatics Retinopathy of Prematurity
BW: Birth weight
GA: Gestational age
MB: Multiple births
MD: Mode of delivery
SD: Standard deviations
CI: Confidence interval
CS: Caesarean section
MLP: Multilayer perceptron
AUCPR: Precision-recall curve area under the curve
AUC: Receiver operating characteristic curve area under the curve
Acc: Accuracy
Sen: Sensitivity
Spec: Specificity
IQR: Interquartile range;
ROC: Receiver operating characteristic
SZEH: Shenzhen Eye Hospital
NICUs: Neonatal Intensive Care Units
